# Elevated Preoperative Serum Alanine Aminotransferase/Aspartate Aminotransferase (ALT/AST) Ratio Is Associated with Better Prognosis in Patients Undergoing Curative Treatment for Gastric Adenocarcinoma

**DOI:** 10.3390/ijms17060911

**Published:** 2016-06-09

**Authors:** Shu-Lin Chen, Jian-Pei Li, Lin-Fang Li, Tao Zeng, Xia He

**Affiliations:** State Key Laboratory of Oncology in South China, Collaborative Innovation Center for Cancer Medicine, Sun Yat-sen University Cancer Center, Guangzhou 510060, China; chenshl@sysucc.org.cn (S.-L.C.); lijianpeisysucc@sina.com (J.-P.L.); linlinf851@aliyun.com (L.-F.L.); liu81219355@163.com (T.Z.)

**Keywords:** ALT/AST ratio (LSR), gastric adenocarcinoma, survival, prognosis

## Abstract

The level of anine aminotransferase/aspartate aminotransferase (ALT/AST) ratio in the serum was often used to assess liver injury. Whether the ALT/AST ratio (LSR) was associated with prognosis for gastric adenocarcinoma (GA) has not been reported in the literature. Our aim was to investigate the prognostic value of the preoperative LSR in patients with GA. A retrospective study was performed in 231 patients with GA undergoing curative resection. The medical records collected include clinical information and laboratory results. We investigated the correlations between the preoperative LSR and overall survival (OS). Survival analysis was conducted with the Kaplan–Meier method, and Cox regression analysis was used to determine significant independent prognostic factors for predicting survival. A *p* value of <0.05 was considered to be statistically significant. A total of 231 patients were finally enrolled. The median overall survival was 47 months. Multivariate analysis indicated that preoperative LSR was an independent prognostic factor in GA. Patients with LSR ≤ 0.80 had a greater risk of death than those with LSR > 0.80. The LSR was independently associated with OS in patients with GA (hazard ratio: 0.610; 95% confidence interval: 0.388–0.958; *p* = 0.032), along with tumor stages (hazard ratio: 3.118; 95% confidence interval: 2.044–4.756; *p* < 0.001) and distant metastases (hazard ratio: 1.957; 95% confidence interval: 1.119–3.422; *p* = 0.019). Our study first established a connection between the preoperative LSR and patients undergoing curative resection for GA, suggesting that LSR was a simple, inexpensive, and easily measurable marker as a prognostic factor, and may help to identify high-risk patients for treatment decisions.

## 1. Introduction

Stomach cancer, or gastric cancer (GC), is a disease in which malignant cells form in the lining of the stomach. The most common type is called adenocarcinoma (GA). Other types of gastric cancer are gastrointestinal carcinoid tumors, gastrointestinal stromal tumors, and lymphomas. GC is one of the most prevalent malignant diseases and the second-most common cause of cancer-related death in the world. Over 70% of new cases and deaths occur in developing countries, with the majority in China [1]. The ratio of men to women is about 2:1. The highest incidence—up to 69 cases per 100,000 people per year—is in men in Northeast Asia [2]. Over the past couple of decades, although advances have been seen in surgical techniques and adjuvant chemotherapy, the prognosis remains dismal [3]. To prolong the survival of GA patients, sensitive and specific factors for classifying cancer risk and predicting survival are always desired in clinics to select patients for tailor treatment.

Serum alanine aminotransferase (ALT) and aspartate aminotransferase (AST) are the main circulating enzymes in the body, are synthesized by the liver, and have half-lives of approximately 18 and 36 h in a healthy young adults, respectively [4]. Transaminases are commonly considered as liver function tests; however, given that, in addition to liver, AST is produced in other tissues, such as heart, muscles, and so on [5,6]. Serum ALT activity has long been used as an inflammatory marker to assess liver injury related to multiple etiologies including hepatitis, tumors, liver cirrhosis, and alcohol consumption, and the level of ALT/AST ratio (LSR) in the serum has been generally accepted as a better predictor of liver injury [7]. Some studies have confirmed that the indexes of liver function could become better prognostic biomarkers [8,9,10,11,12]. However, there are no studies that have evaluated the association between the preoperative serum LSR and survival in GA. Thus, the prognostic value of the LSR in patients with GA is not clear. In this study, we performed a large-scale retrospective cohort analysis to examine the association between LSR and survival in GA patients who are undergoing curative treatment.

## 2. Results

### 2.1. Patients

A total of 231 eligible patients were identified from January 2008 to December 2015. The clinicopathologic characteristics of the 231 GA patients in this study are described in Table 1. There were 160 men (69.3%) and 71 women (30.7%) with a median age of 56 years (range, 23–79 years). Of these, 29 (12.6%) were stage I, 52 (22.5%) were stage II, 94 (40.7%) were stage III, and 56 (24.2%) were stage IV. At the time of last follow-up, the median overall survival (OS) was 47 months.

### 2.2. Serum ALT/AST Ratio (LSR) and Survival

**Figure 1:**
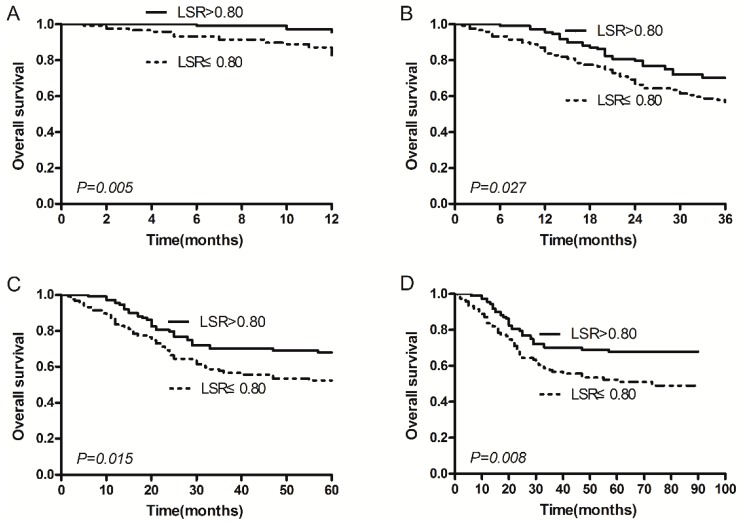
Kaplan–Meier survival curves depict survival curve according to the ALT/AST ratio (LSR). (**A**) One-year survival according to LSR > 0.80/LSR ≤ 0.80; (**B**) three-year survival curve according to LSR > 0.80/LSR ≤ 0.80; (**C**) five-year survival curve according to LSR > 0.80/LSR ≤ 0.80; and (**D**) the overall survival (OS) curve according to LSR > 0.80/LSR ≤ 0.80.

Comparing multiple Kaplan-Meier curves, a significant result from the log rank test indicated that LSR > 0.80 was associated with better OS (*p* = 0.008; Figure 1D). The mean OS times of patients with LSR > 0.80 and LSR ≤ 0.80 were 68.2 and 55.5 months, respectively, and one-year survival rate (*p* = 0.005; Figure 1A), three-year survival rate (*p* = 0.027; Figure 1B), and five-year survival rate (*p* = 0.015; Figure 1C) were 95.5% *vs.* 84.5%, 70.1% *vs.* 56.7%, and 67.8% *vs.* 52.3%, respectively. In the univariate analysis, variables including pathological stage (*p* < 0.001), lymph node metastasis (*p* < 0.001), distant metastases (*p* < 0.001), location (*p* = 0.021), tumor size (*p* < 0.001), serous infiltration (*p* < 0.001), albumin (ALB) (*p* = 0.020), LSR (*p* = 0.009), CA72-4 (*p* = 0.001), Fibrinogen (Fbg) (*p* = 0.049), Glasgow Prognostic Score (GPS) (*p* = 0.035), and p53 (*p* = 0.045) were significant risk factors for GA survival (Table 2), whereas sex (*p* = 0.481), age (*p* = 0.187), smoking (*p* = 0.313), family history (*p* = 0.566), ALT (*p* = 0.337), AST (*p* = 0.869), C-reaction protein (CRP) (*p* = 0.160), CA19-9 (*p* = 0.085), Carcino-embryonic antigen (CEA) (*p* = 0.060), and ABO blood group (*p* = 0.115) were not (Table 2).

The variables identified as predictors of OS in the univariate analysis were then entered into multivariate Cox regression analysis (Table 2). The results showed LSR (HR 0.610; 95% CI 0.388–0.958, *p* = 0.032), TNM stage (HR 3.118; 95% CI 2.044–4.756, *p* < 0.001), and distant metastases (HR 1.957; 95% CI 1.119–3.422, *p* = 0.019) were independent predictive factors for gastric cancer. Patients with LSR ≤ 0.80 had a greater risk of death than those with LSR > 0.80. However, lymph node metastasis (HR 0.831; 95% CI 0.554–1.247; *p* = 0.371), location (HR 0.852; 95% CI 0.646–1.123; *p* = 0.255), tumor size (HR 1.180; 95% CI 0.752–1.852; *p* = 0.472), serous infiltration (HR 0.972; 95% CI 0.708–1.334; *p* = 0.860), ALB (HR 0.776; 95% CI 0.247–2.438; *p* = 0.664), CA72-4 (HR 1.479; 95% CI 0.941–2.325; *p* = 0.090), Fbg (HR 1.070; 95% CI 0.669–1.713; *p* = 0.778), GPS (HR 0.929; 95% CI 0.431–2.004; *p* = 0.851), and p53 (HR 1.128; 95% CI 0.917–1.387; *p* = 0.255) were not significant predictive factors in multivariate analysis.

### 2.3. The Relationship between the ALT/AST Ratio and Clinicopathologic Characteristics in GA Patients

Further analysis of the relation among the two groups (LSR > 0.80 and LSR ≤ 0.80) and other prognostic indicators for GA (Table 3). LSR was associated with sex (*p* = 0.023), tumor sizes (*p* = 0.041), ALB (*p* = 0.020), ALT (*p* < 0.001), AST (*p* = 0.004), CA19-9 (*p* = 0.008), and CRP (*p* = 0.025). However, LSR did not show any significant association with age (*p* = 0.148), smoking behavior (*p* = 0.141), family history (*p* = 0.515), TNM stage (*p* = 0.425), lymph node metastasis (*p* = 0.168), distant metastases (*p* = 0.294), tumor location (*p* = 0.247), serous infiltration (*p* = 0.055), CA72-4 (*p* = 0.600), CEA (*p* = 0.793), Fbg (*p* = 0.694), GPS (*p* = 0.054), p53 (*p* = 0.075), and blood type (*p* = 0.736).

## 3. Discussion

Gastric cancer is the most common cancer and the second leading cause of cancer-related death in the world. Factors that may increase risk of gastric cancer include oxidative stress, DNA damage, *Helicobacter pylori* infection, and so on. Inflammation and *H. Pylori* infection induce oxidative stress, which leads to DNA damage, impairs immune function, extracellular signal-regulated kinase (ERK) activations and p53 over expression, which are related to cancer development [13].

The liver is the largest solid organ and plays a major role in metabolism with numerous functions in the human body. ALT and AST are the major critical enzymes in the biological processes [14]. The synthesized ALT and stored AST changes in serum levels have become diagnostic tools and markers for assessing the liver function [15]. Reports have suggested that their levels increase in different hepatic injures, such as hepatitis and cirrhosis induced by alcohol, drugs, viruses, and also under oxidative stress [16]. The liver could easily be exposed to internal stimuli which produce reactive oxygen species. The oxidative stress could damage the liver cells. The levels of ALT/AST in the serum have been generally accepted as a better predictor of liver injury [7]. Oxidative stress and inflammation are related to gastric cancer development; at the same time, oxidative stress and inflammation could also lead to damaged liver cells. With the development of tumor biology, there is growing evidence that the presence of a systemic inflammatory response is linked to poor survival in patients with different types of cancers [17,18]. Thus, our aim was to investigate the prognostic value of the preoperative LSR in patients with GA.

This study is mainly focused on the association between clinicopathological parameters and prognosis in gastric cancer. Previous studies have shown that serum albumin, CRP, TNM stage, serum globulin HER-2, CA72-4, and GPS were associated with OS in GC, and they could also predict prognostic factors for survival in gastric cancer [9,10,11,17,19,20]. In our study, only TNM stage, lymph node metastasis, distant metastases, tumor size, serous infiltration, ALB, LSR, CA72-4, Fbg, and p53 were shown to be predictors of OS in univariate analyses. However, the following multivariate analysis showed only LSR was independently associated with OS in patients with GA (*p* = 0.032), along with tumor stages (*p* < 0.001) and distant metastases (*p* = 0.019). Patients with LSR ≤ 0.80 were associated with significantly worse survival than those with LSR > 0.80. In our study, GPS and CRP were not as independent prognostic factors for survival in gastric cancer. We explained the results and the factors with *p* values <0.1 in the univariate analysis were then performed using multivariate Cox regression analysis to identify independent predictors of OS. The factors we studied were more than other researchers did and the prognostic factors were more comprehensive.

Until now, many researchers have reported inflammation and oxidative stress were especially important for cancer progress. The reasons that inflammation and oxidative stress are related to gastric cancer is as follows: (1) tumor progression and metastasis comprise a cascade of steps which involve tumorigenic factors (inflammation, oxidative stress) which participate in the process [21,22,23]; (2) inflammatory and chronic oxidative stress leads to generating oxygen free radicals, which have been shown to stimulate cancer initiation, promotion, and progression [21]; (3) the gastrointestinal tract could easily be exposed to external and internal stimuli which produce ROS [24]; (4) systemic inflammation showed correlation with therapy response.

Serum transaminases were used to assess the stage of fibrosis, liver function, and prediction of prognosis for patients with chronic hepatitis [25]. Ronnie [26] reported an increased serum AST level was an independent adverse prognostic factor in patients with hepatocellular carcinoma associated with HBV-related cirrhosis. They considered the elevated transaminases were associated with hepatitis activity in the cirrhotic liver; this may increase the risk of recurrence. Shen [27] also found preoperative AST was an independent prognostic factor for hepatitis B-induced hepatocellular carcinoma after hepatic resection. Recent studies demonstrated that proliferating cancer cells show aerobic glycolysis, as well as increased glutamine metabolism, to maintain nucleotide biosynthesis and nonessential amino acids, which are catalyzed by transaminases [28,29]. The exact explanation for the decreased death rate in patients with a preoperatively-elevated ALT/AST ratio remain elusive.

The major limitations of this study are as follows: (1) this study was a retrospective design, single-center site, and lacking measurements of *H. pylori* (HP) infection, since we could not obtain information from most studies regarding infection with HP, a strong risk factor for gastric cancer [30]; and (2) our study found that LSR was an independent prognostic factor in patients with GA, but we did not reveal the mechanisms of how LSR influences the patients’ survival times. Further studies may aim at investigating what the role LSR plays in the prognosis of GA. Despite these limitations, we report the first study of the potential prognostic value of the preoperatively-evaluated LSR in patients of gastric cancer.

In recent years, “liquid biopsies” have attracted increasing interest for noninvasive cancer diagnosis, prognostis, and monitoring of treatment response. Several studies have provided proof-of-principle data related to several circulating markers that could be used for cancer prognosis. These blood-based biomarkers include circulating tumor cells, DNA-, RNA-, and protein-based markers. We evaluated the association between the circulating protein-based markers and survival in GA and found LSR was an independent predictor of poor survival in GA, thus, it is a new prognostic marker in gastric cancer.

In conclusion, our first report LSR was an independent predictor of poor survival in gastric cancer. The following study programs should validate this association and elucidate the mechanisms by which LSR affects the risk of prognosis in gastric cancer. In a word, preoperative serum LSR is a simple, convenient, and low-cost prognostic marker in gastric adenocarcinoma patients undergoing curative treatment.

## 4. Materials and Methods

### 4.1. Patients

A total of 231 histologically-proven gastric adenocarcinoma patients at the Sun Yat-sen University Cancer Center who were treated with undergoing curative treatment from 2008 to 2015 were enrolled in this study. Collection and recording of the patient's clinical data, including sex, age, smoking, family history, location, ABO blood group, tumor size, serous infiltration, lymph node metastasis, CA72-4, CA19-9, CEA, ALB, C-reactive protein, p53, ALT, AST, ALT/AST, Fibrinogen (Fbg), GPS score, TNM stage, diagnosis, treatment, and follow-up results was conducted. All procedures performed were in accordance with the ethical standards of the national regulations and with the 1964 Helsinki Declaration and its later amendments. Our study was approved by the Sun Yat-sen University Cancer Center research ethics committee. The study was approved by the Ethics Committee of Sun Yat-Sen University Cancer Center, and all patients signed an informed consent before inclusion in the study. The inclusion criteria as following: (1) all patients were histologically confirmed as having stage I to IV adenocarcinoma of the stomach depending on postoperative histological specimen; (2) clinical data were complete; (3) good performance status; (4) undergoing curative treatment; (5) patients with other malignant tumors; and (6) no liver dysfunction. All patients were followed up until death or 30 October 2015, if still alive.

### 4.2. Laboratory Measurements

Fbg was measured by coagulation method using Sysmex CA7000 System (Sysmex, Kobe, Japan). ALT, AST, ALB, CRP were measured by Hitachi 7600 Automatic Analyzer (Tokyo, Japan). CA19-9, CA72-4, CEA were measured using electrochemiluminescence immunoassay (Roche COBAS-E-602, Shanghai, China). The value of Fbg, ALT, AST, ALB, CRP, CA19-9, CA72-4, CEA testing one day before pre-operation was recorded. The LSR was defined as the serum ALT level divided by the serum AST level. The GPS was calculated by CRP and ALB using standard thresholds (>10 mg/L for CRP and ≤35 g/L for ALB). Patients with both a CRP level >10 mg/L and an ALB level ≤35 g/L were categorized as having a score of 2. Patients with only one of these abnormalities were categorized as having a score of 1. Patients with neither of these abnormalities were categorized as having a score of 0 [31]. p53 evaluation was carried out according to the data available in the literature [32]. Accordingly, they were assigned scores of 0, +1, +2, and +3 according to their immune-reactivity.

### 4.3. Statistical Analysis

Data was summarized with the number of subjects and mean value, and the cutoff value of preoperative Fbg, ALT, AST, CRP, CA19-9, CA72-4, CEA, and LSR were estimated by median. Overall survival (OS) was calculated from the date of surgery to the date of death or last follow-up. Statistical analysis was performed by SPSS software, version 19.0 (SPSS Inc., Chicago, IL, USA). The Chi-square test was used to compare categorical variables. Multivariate Cox regression was used to perform survival analysis in order to estimate the variables for survival. Results of the Cox regression analyses were reported with hazard ratios (HR), together with the corresponding 95% confidence intervals (CI). Survival curves were constructed according to the Kaplan-Meier method and compared using the log-rank test. All factors significant in univariate analyses were entered into a multivariate analysis. Two-tailed *p* values <0.05 were considered significant.

## Figures and Tables

**Table 1:** Clinical and laboratory characteristics of 231 patients associated with overall survival (OS).

Patient Characteristics	No. of Patients (%)	OS (Months) Mean (95% CI)	*p*-Value Log-Rank
Sex			
Male	160 (69.3%)	62.5 (57.0–67.9)	0.478
Female	71 (30.7%)	59.7 (51.4–68.1)	
Age (years)			
≤56	118 (51.1%)	64.4 (58.2–70.6)	0.183
>56	113 (48.9%)	58.8 (52.1–65.6)	
Smoking Behaviour			
Yes	64 (27.7%)	65.5 (57.1–73.9)	0.308
No	167 (72.3)	60.4 (54.9–65.8)	
Family History			
Yes	23 (10.0%)	63.3 (50.3–76.3)	0.563
No	208 (90.0%)	61.5 (56.4–66.4)	
Pathological Stage ^a^			
I	29 (12.6%)	86.8 (84.4–89.1)	<0.001
II	52 (22.5%)	85.9 (82.1–89.8)	
III	94 (40.7%)	57.9 (50.9–64.9)	
IV	56 (24.2%)	29.6 (21.7–37.5)	
Lymph Node Metastasis (N)			
N0	17 (7.3%)	74.9 (62.2–87.6)	<0.001
N1	38 (16.5%)	70.8 (60.1–80.5)	
N2	156 (67.5%)	60.3 (54.9–65.8)	
N3	20 (8.7%)	35.4 (19.7–51.1)	
Distant Metastases			
Yes	39 (16.9%)	25.4 (17.7–33.2)	<0.001
No	192 (83.1%)	68.7 (64.1–73.3)	
Tumor Location ^a^			
Upper	49 (21.2%)	51.3 (41.7–60.8)	0.062
Middle	61 (26.4%)	58.4 (49.9–66.9)	
Lower	121 (52.4%)	66.7 (60.5–72.9)	
Maximun Tumor Diameter (cm)			
≤5	144 (62.3%)	70.1 (64.9–75.4)	<0.001
>5	87 (37.7%)	48.1 (40.4–55.7)	
Serous Infiltration			
S0	44 (19.1%)	86.0 (81.8–90.1)	<0.001
S1	37 (16.0%)	63.9 (52.2–75.5)	
S2	100 (43.3%)	58.3 (51.5–65.2)	
S3	50 (21.6%)	43.0 (33.3–52.7)	
ALB (g/L)			
≤35	21 (9.1)	41.7 (26.9–56.4)	0.017
>35	210 (90.9%)	63.7 (58.9–68.4)	
ALT (U/L)			
≤14.6	114 (49.4%)	59.3 (52.9–65.7)	0.334
>14.6	117 (50.6%)	64.1 (57.6–70.6)	
AST (U/L)			
≤18.5	116 (50.2%)	61.1 (54.7–67.5)	0.868
>18.5	115 (49.8%)	62.4 (55.8–68.9)	
LSR (ALT/AST)			
≤0.80	117 (50.6%)	55.5 (48.9–62.1)	0.008
>0.80	114 (49.4%)	68.2 (62.1–74.3)	
CRP (mg/L)			
≤1.56	116 (50.2%)	65.4 (59.2–71.6)	0.156
>1.56	115 (49.8%)	58.0 (51.4–64.7)	
CA19-9 (U/mL)			
≤10.97	117 (50.6%)	66.2 (59.8–72.6)	0.081
>10.97	114 (49.4%)	57.5 (51.1–64.0)	
CA72-4 (U/mL)			
≤1.8	116 (50.2%)	69.7 (63.7–75.5)	<0.001
>1.8	115 (49.8%)	53.9 (47.2–60.5)	
CEA (ng/mL)			
≤2.17	117 (50.6%)	66.2 (60.0–72.6)	0.057
>2.17	114 (49.4%)	57.2 (50.7–63.7)	
Fbg (g/L)			
≤2.82	116 (50.2%)	66.6 (60.4–72.8)	0.046
>2.82	115 (49.8%)	56.8 (50.2–63.4)	
GPS			
0	193 (83.6%)	64.3 (59.4–69.1)	0.066
1	34 (14.7%)	50.1 (37.0–63.1)	
2	4 (1.7%)	43.5 (13.8–73.2)	
p53			
0	56 (24.2%)	59.7 (50.2–69.3)	0.018
+1	75 (32.5%)	70.3 (63.4–77.2)	
+2	34 (14.7%)	59.0 (47.2–70.7)	
+3	66 (28.6%)	52.9 (44.1–61.6)	
Blood Type			
A	63 (27.3%)	57.2 (48.3–66.1)	0.412
B	16 (6.9%)	51.9 (37.5–66.2)	
AB	57 (24.7%)	59.2 (50.2–68.2)	
O	95 (41.1%)	66.3 (59.3–73.2)	

^a^ According to the 7th UICC-TNM classification; ALT, anine aminotransferase; AST, aspartate aminotransferase; Fbg, Fibrinogen; ALB, albumin; CRP, C-reaction protein; CEA, carcino-embryonic antigen; GPS, Glasgow Prognostic Score.

**Table 2:** Univariate and multivariate analysis of OS.

Patient Characteristics	Univariate Analysis HR (95% CI)	*p*-Value	Multivariate Analysis HR (95% CI)	*p*-Value
Sex	0.853 (0.549–1.326)	0.481	-	-
Age (years)	1.326 (0.872–2.018)	0.187	-	-
Smoking Behaviour	0.777 (0.476–1.268)	0.313	-	-
Family History	0.808 (0.391–1.672)	0.566	-	-
Pathological Stage	3.666 (2.694–4.988)	<0.001	3.118 (2.044–4.756)	<0.001
Lymph Node Metastasis (N)	1.988 (1.375–2.874)	<0.001	0.831 (0.554–1.247)	0.371
Distant Metastases	5.286 (3.360–8.316)	<0.001	1.957 (1.119–3.422)	0.019
Tumor Location	0.746 (0.581–0.957)	0.021	0.852 (0.646–1.123)	0.255
Maximun Tumor Diameter (cm)	2.528 (1.660–3.848)	<0.001	1.180 (0.752–1.852)	0.472
Serous Infiltration	2.038 (1.587–2.618)	<0.001	0.972 (0.708–1.334)	0.860
ALB (g/L)	0.485 (0.264–0.892)	0.020	0.776 (0.247–2.438)	0.664
ALT (U/L)	0.814 (0.535–1.239)	0.337	-	-
AST (U/L)	0.965 (0.636–1.466)	0.869	-	-
LSR (ALT/AST)	0.565 (0.368–0.868)	0.009	0.610 (0.388–0.958)	0.032
CRP (mg/L)	1.351 (0.888–2.057)	0.160	-	-
CA19-9 (U/mL)	1.454 (0.950–2.224)	0.085	0.710 (0.442–1.139)	0.155
CA72-4 (U/mL)	2.035 (1.318–3.144)	0.001	1.479 (0.941–2.325)	0.090
CEA (ng/mL)	1.501 (0.983–2.293)	0.060	1.331 (0.811–2.184)	0.258
Fbg (g/L)	1.530 (1.002–2.338)	0.049	1.070 (0.669–1.713)	0.778
GPS	1.565 (1.033–2.373)	0.035	0.929 (0.431–2.004)	0.851
p53	1.212 (1.005–1.462)	0.045	1.128 (0.917–1.387)	0.255
Blood Type	0.875 (0.741–1.033)	0.115	-	-

**Table 3:** Relationship between the LSR and clinicopathologic characteristics.

Patient Characteristics	LSR ≤ 0.80 (*n* = 117)	LSR > 0.80 (*n* = 114)	*p*-Value
Sex			
Male	73	87	0.023
Female	44	27	
Age (years)			
≤56	54	64	0.148
>56	63	50	
Smoking Behavior			
Yes	90	77	0.141
No	27	37	
Family History			
Yes	107	101	0.515
No	10	13	
Pathological Stage			
I	13	16	0.425
II	23	29	
III	48	46	
IV	33	23	
Lymph Node Metastasis (N)			
N0	7	10	0.168
N1	22	16	
N2	74	82	
N3	14	6	
Distant Metastases			
Yes	94	98	0.294
No	23	16	
Tumor Location			
Upper	27	22	0.247
Middle	35	26	
Lower	55	66	
Maximum Tumor Diameter (cm)			
≤5	65	79	0.041
>5	52	35	
Serous Infiltration			
S0	16	28	0.055
S1	23	14	
S2	48	52	
S3	30	20	
ALB (g/L)			
≤35	16	5	0.020
>35	101	109	
ALT (U/L)			
≤14.6	94	20	<0.001
>14.6	23	94	
AST (U/L)			
≤18.5	70	46	0.004
>18.5	47	68	
CRP (mg/L)			
≤1.56	50	66	0.025
>1.56	67	48	
CA19-9 (U/mL)			
≤10.97	49	68	0.008
>10.97	68	46	
CA72-4 (U/mL)			
≤1.8	61	55	0.600
>1.8	56	59	
CEA (ng/mL)			
≤2.17	58	59	0.793
>2.17	59	55	
Fbg (g/L)			
≤2.82	57	59	0.694
>2.82	60	55	
GPS			
0	91	102	0.054
1	23	11	
2	3	1	
p53			
0	23	33	0.075
+1	42	33	
+2	13	21	
+3	39	27	
Blood type			
A	35	28	0.736
B	28	29	
AB	9	7	
O	45	50	
